# Activation of Ca^2+^‐AMPK‐mediated autophagy by ginsenoside Rg3 attenuates cellular senescence in human dermal fibroblasts

**DOI:** 10.1002/ctm2.521

**Published:** 2021-08-09

**Authors:** Dasol Kim, Kyeong Eun Yang, Dong Won Kim, Hui‐Yun Hwang, Jinyoung Kim, Jong‐Soon Choi, Ho Jeong Kwon

**Affiliations:** ^1^ Chemical Genomics Leader/Global Research Laboratory Department of Biotechnology College of Life Science and Biotechnology Yonsei University Seoul Republic of Korea; ^2^ Bio‐Chemical Analysis Group Center for Research Equipment Korea Basic Science Institute Daejeon Republic of Korea; ^3^ Research Center for Materials Analysis Korea Basic Science Institute Daejeon Republic of Korea

Dear Editor,

Cellular senescence is a multifaceted process where permanent cell cycle arrest occurs under stresses, indicating the hallmark of aging decline in organisms.^1^ Ginsenoside Rg3 (Rg3) has been reported to promote rejuvenation of replicatively aged human dermal fibroblasts (HDFs) via regulation of reactive oxygen species (ROS) and Akt‐mTOR‐Sirtuin signaling, respectively.^2^, ^3^ However, little study has examined the relationship between the regulation of senescence by Rg3 and autophagy, even though an increasing number of reports have assessed the role of Rg3 in autophagy regulation.^4^ Therefore, the present study investigated the role of autophagy in Rg3‐induced senescence retention in HDFs.

As reported previously,^3^ Rg3 reversed cellular replicative senescence in HDFs, exhibiting reduced SA‐β‐gal activity and attenuated TP53 and CDKN1A expression levels (Figures 1A, B and S1A–C). To examine whether Rg3 can attenuate cellular senescence in the skin tissue *in vivo*, where aging skin negatively affects tissue repair, wound healing was analyzed in 4‐month‐old (young) and 18‐month‐old (old) mice. Immunohistochemistry staining of proliferating cell nuclear antigen and Ki‐67 did not reveal a significant difference between young mice given either treatment; however, old Rg3‐treated mice exhibited remarkably more positively stained cells than old control mice (Figure 1C, D). In addition, based on the antioxidant activity of Rg3 previously reported in HDFs,^2^ we investigated the role of the Rg3‐induced antioxidant effect in cellular senescence in HDFs. Rg3 treatment not only reduced intracellular ROS level (Figure 1E), but also reversed H_2_O_2_‐induced senescence (Figures 1F and S2), suggesting that the antioxidant activity of Rg3 can attenuate cellular senescence.

**FIGURE 1:**
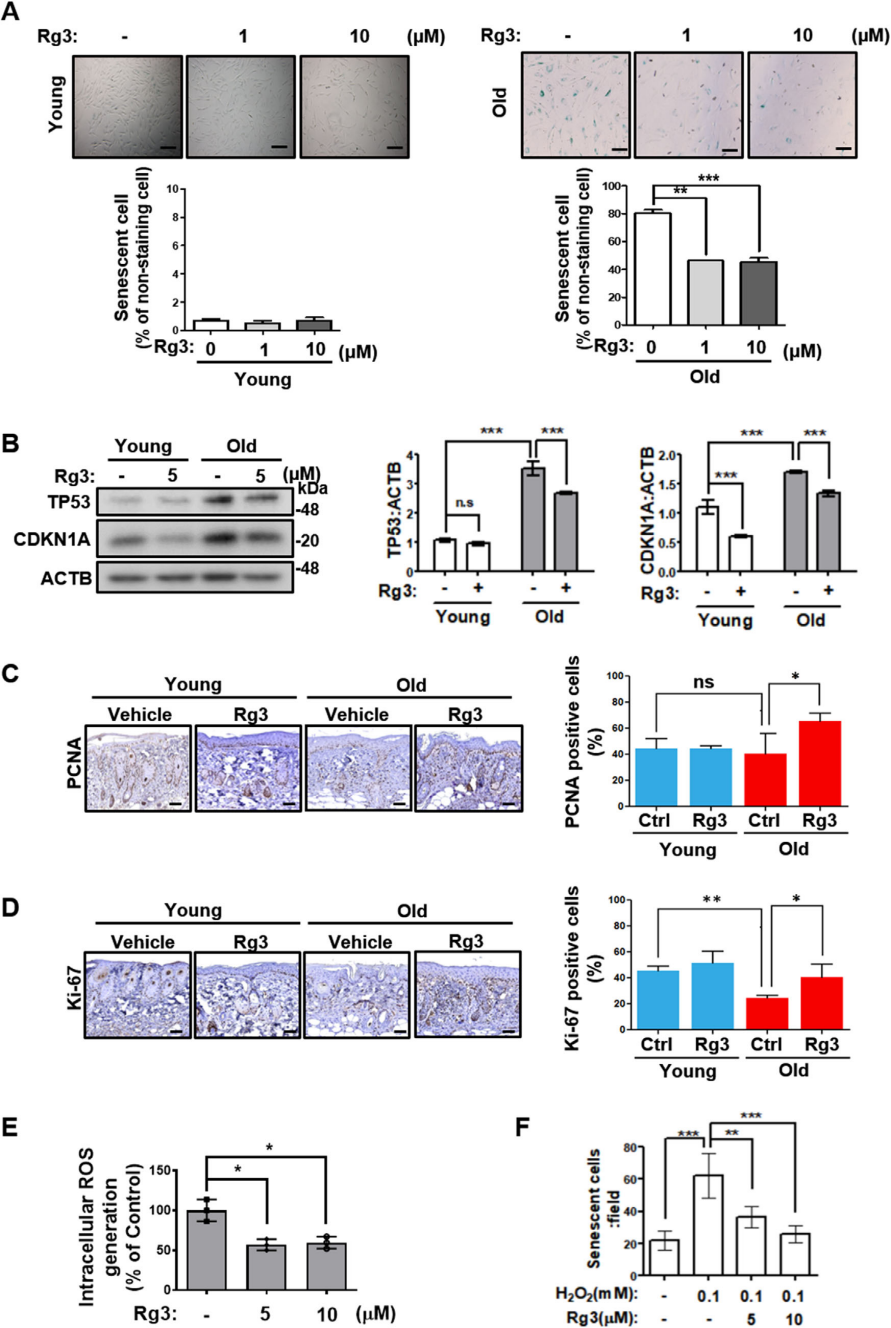
GinsenosideRg3 improves replicative aging declines in skin through attenuating cellular oxidative stress. (A) Young and old human dermal fibroblast (HDF) cells treated with Rg3 (1 or 10 μM) for 48 h and then processed for the senescence‐associated β‐galactosidase (SA‐β‐gal) assay. Representative images (upper) and cell counting per fields (below). Graph shows the means ± SD (*n*  =  3). Scale bar, 200 μm. (B) Young and old HDFs treated with 5 μM Rg3 for 24 h. Cell extracts were subjected to western blotting. Representative images (left) and immunoblot band intensity normalized to ATCB (right). The graph shows the means ± SD (*n*  =  3). (C, D) Excised wounds were made in the dorsal skin of 4‐month‐old (young) and 18‐month‐old (old) C57BL6 mice. The mice were treated with the vehicle or 100 μM Rg3 once every 2 days for a week. Skin tissues were sectioned and assessed by immunohistochemistry using proliferating cell nuclear antigen (PCNA) (C) and Ki‐67 (D) antibodies. Representative images (left) and percentage of PCNA or Ki‐67 marker positive cells (right). Graphs show the means ± SD (*n*  =  6). Scale bar, 100 μm. (E) ROS levels measured in Rg3‐treated old HDFs. Cells were stained with dichlorofluorescein diacetate, fixed, and immediately analyzed using a multianalytic validation system. The graph shows the means ± SD (*n*  =  3). (F) Young HDF cells treated with Rg3 (5 or 10 μM) with 0.1 mM H_2_O_2_ for 48 h. Cells were processed for the SA‐β‐gal assay. The graph shows the number of cell counts per field with means ± SD (*n*  =  4). Statistical significance was assessed by one‐way ANOVA with Tukey's post‐hoc test. ***p < 0.001; **p < 0.01; *p < 0.05

Since activation of the NRF2 pathway has been identified as a major antioxidant mechanism, we explored the relevancy of Rg3's antioxidant effect to NRF2 signaling. Rg3‐treated HDF cells exhibited increased levels of NRF2 protein and its downstream target gene, *HMOX1* (Figure 2A). Moreover, Rg3‐treated aging cells showed more sensitive response against H_2_O_2_‐derived oxidative stress with increased HMOX1 level, whereas nontreated senescent cells exhibited poor response (Figures 2B and S3). Recent studies have revealed that autophagy can enhance NRF2 stability, through promoting SQSTM1‐sequestration‐induced KEAP1 degradation.^5^ Therefore, we investigated the role of autophagy in Rg3‐induced NRF2 activation. Notably, HMOX1 induction in Rg3‐primed HDFs was abolished upon treatment with the autophagosome formation inhibitor 3‐methyladenine (Figure 2C). In addition, given that the SQSTM1 sequestration of KEAP1 is triggered after SQSTM1 phosphorylation,^6^ the phosphorylation state on serine 351 of SQSTM1 was explored, resulting in increased phosphorylation upon Rg3 treatment (Figure 2D). Moreover, the abolished efficacy of Rg3 on the downregulation of SA‐β‐gal activity upon autophagy inhibition indicated the pivotal role of autophagy in Rg3‐mediated senescence regulation (Figure S4A). Similar to the results of many studies reporting decreased autophagy in aged cells,^7^ HDF cells in this study exhibited the inhibition of autophagy flux when under replicative senescence (Figure S4B).

**FIGURE 2:**
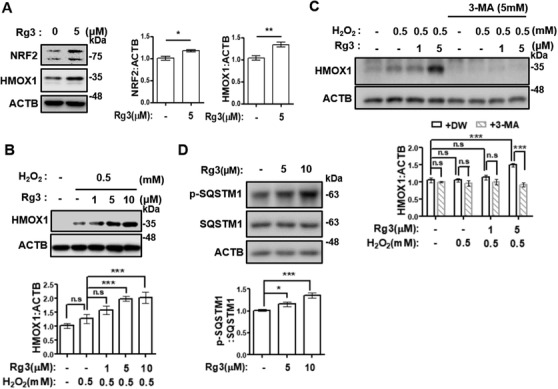
Rg3 enhances NRF2 signaling in senescent cells through promoting autophagy. (A) Old HDFs were treated with 5 μM Rg3 for 72 h. (B) Old HDFs were treated with Rg3 (1, 5, or 10 μM) for 48 h, then exposed to 0.5 mM H_2_O_2_ for 4 h. (C) Old HDFs were treated with Rg3 (1 or 5 μM) with or without 3‐methyladenine for 48 h, and then exposed to 0.5 mM H_2_O_2_ for 4 h. (D) Old HDFs were treated with Rg3 (5 or 10 μM) for 1 h. Cell extracts from each experiment (A–D) were subjected to immunoblotting. Representative images (left or upper) and immunoblot band intensity normalized to ACTB (right or below). The graphs show the means ± SD (*n*  =  3). Statistical significance was assessed by Student's *t*‐test or one‐way ANOVA with Tukey's post‐hoc test. ***p < 0.001; **p < 0.01; *p < 0.05

A recent study revealed that AMPK induces the phosphorylation of SQSTM1 at serine 351 to enhance NRF2 activation.^8^ As we previously identified AMPK activation by Rg3, the possible role of AMPK in Rg3‐mediated NRF2 activation was investigated. AMPK knockdown abolished HMOX1 expression level upon Rg3 treatment (Figure 3A). Notably, Rg3‐induced SQSTM1 phosphorylation was also diminished by the pharmacological inhibition of AMPK using Compound C (CC) (Figure 3B). Moreover, Rg3 treatment induced both BECN1 phosphorylation at serine 93 and LC3 conversion, whereas AMPK inhibitor treatment reverted (Figure 3C–E). In addition, tandem fluorescence‐tagged LC3 (mRFP‐EGFP‐LC3) indicated that Rg3‐treated HDFs increased the number of autolysosomes (red puncta), but Bafilmycin A1 treatment in Rg3‐treated HDFs yielded remarkably accumulated autophagosomes (yellow puncta), indicating that Rg3 enhances autophagosome formation and autophagy flux (Figure 3F). By contrast, AMPK knockdown suppressed Rg3‐induced autophagy (Figure 3G). These results demonstrated that Rg3 requires AMPK signaling to induce autophagy and NRF2 activation.

**FIGURE 3:**
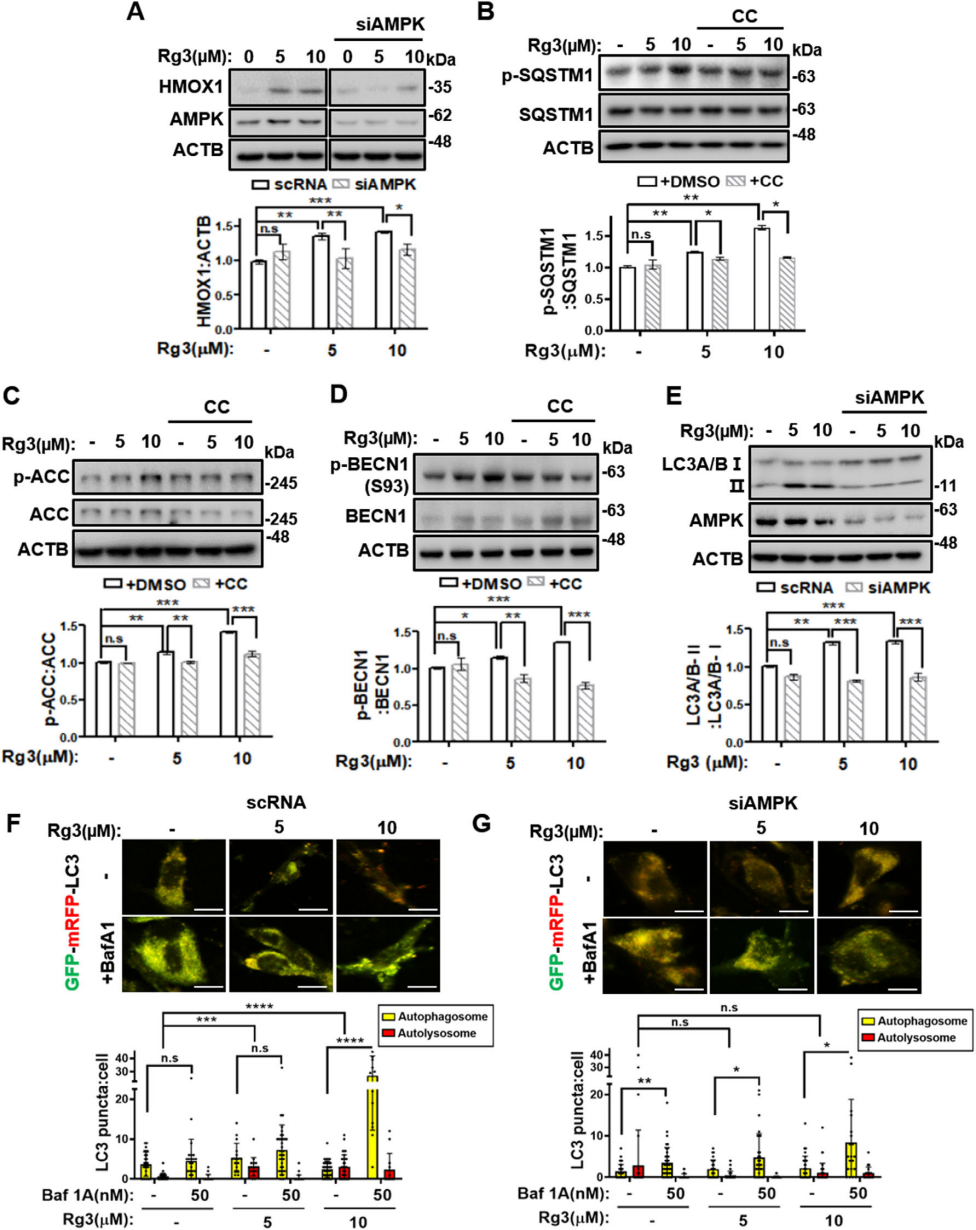
Rg3 induces autophagy in an AMPK signaling–dependent manner. (A) Old HDFs were transfected with siRNA targeting AMPK for 24 h, and then treated with Rg3 (5 or 10 μM) for 48 h. (B) Mid‐old (passage 26) HDFs were treated with (upper) and immunoblot band intensity normalized to ACTB (lower). The graph shows the means ± SD (*n*  =  3). (C) Mid‐old (passage 27) HDFs were treated with Rg3 (5 or 10 μM) for 1 h, with or without pretreatment of 1 μM Compound C (CC). (D) Old HDFs were treated with Rg3 (5 or 10 μM) for 1 h, with or without pretreatment of 1 μM CC. (E) Old HDFs were transfected with siRNA targeting AMPK for 24 h, and then treated with Rg3 (5 or 10 μM) for 24 h. Cell extracts from each experiment (A–E) were subjected to immunoblotting. Representative images (upper) and immunoblot band intensity normalized to ACTB (below). Graphs show the means ± SD (*n*  =  3). (F, G) Old HDFs transfected with double‐tagged GFP‐mRFP‐LC3 and nontargeting scrambled RNA (scRNA) or siRNA targeting AMPK were first treated with Rg3 (5 or 10 μM) for 44 h, and then treated with 50 nM bafilomycin A1 (Baf A1) for 4 h. Representative images of cells under confocal microscopy (upper) and graphs showing the number of yellow (autophagosome) and red (autolysosome) puncta per cell (below). Scale bar, 20 μm. Graphs show the means ± SD (*n* ≥ 25). Statistical significance was assessed by one‐way ANOVA with Tukey's post‐hoc test. ***p < 0.001; **p < 0.01; *p < 0.05

AMPK can be activated by two distinct signaling pathways: the AMP‐LKB1‐mediated pathway and the Ca^2+^‐CAMKK2‐mediated pathway.^9^ The activation of AMPK by Rg3 was blocked by STO‐609 (CAMKK2 inhibitor), similar to ionomycin (Ca^2+^ ionophore); however, activation by itraconazole (ATP‐depleting agent) was not reversed (Figure 4A). These results indicate that Rg3 promotes AMPK signaling activation in a Ca^2+^‐dependent manner. Moreover, ethylene glycol tetraacetic acid (EGTA, extracellular Ca^2+^ chelator) pretreatment abolished Rg3‐induced AMPK activation. By contrast, itraconazole‐induced AMPK was not reverted. Ionomycin most dramatically induced AMPK activation; however, this was partially reverted by EGTA pretreatment. Glycyl‐L‐phenylalanine‐β‐naphthylamide (GPN, ER, and Lysosome Ca^2+^‐releasing agent)‐induced AMPK activation did not exhibit any perturbation by EGTA (Figure 4B). Rg3 still activated AMPK signaling with GPN pretreatment but did not activate with EGTA (Figure S5A). These results demonstrated that extracellular Ca^2+^ is required for Rg3, suggesting a new relevancy of Ca^2+^ channels located in the plasma membrane for Rg3‐induced AMPK signaling activation in HDFs.

**FIGURE 4:**
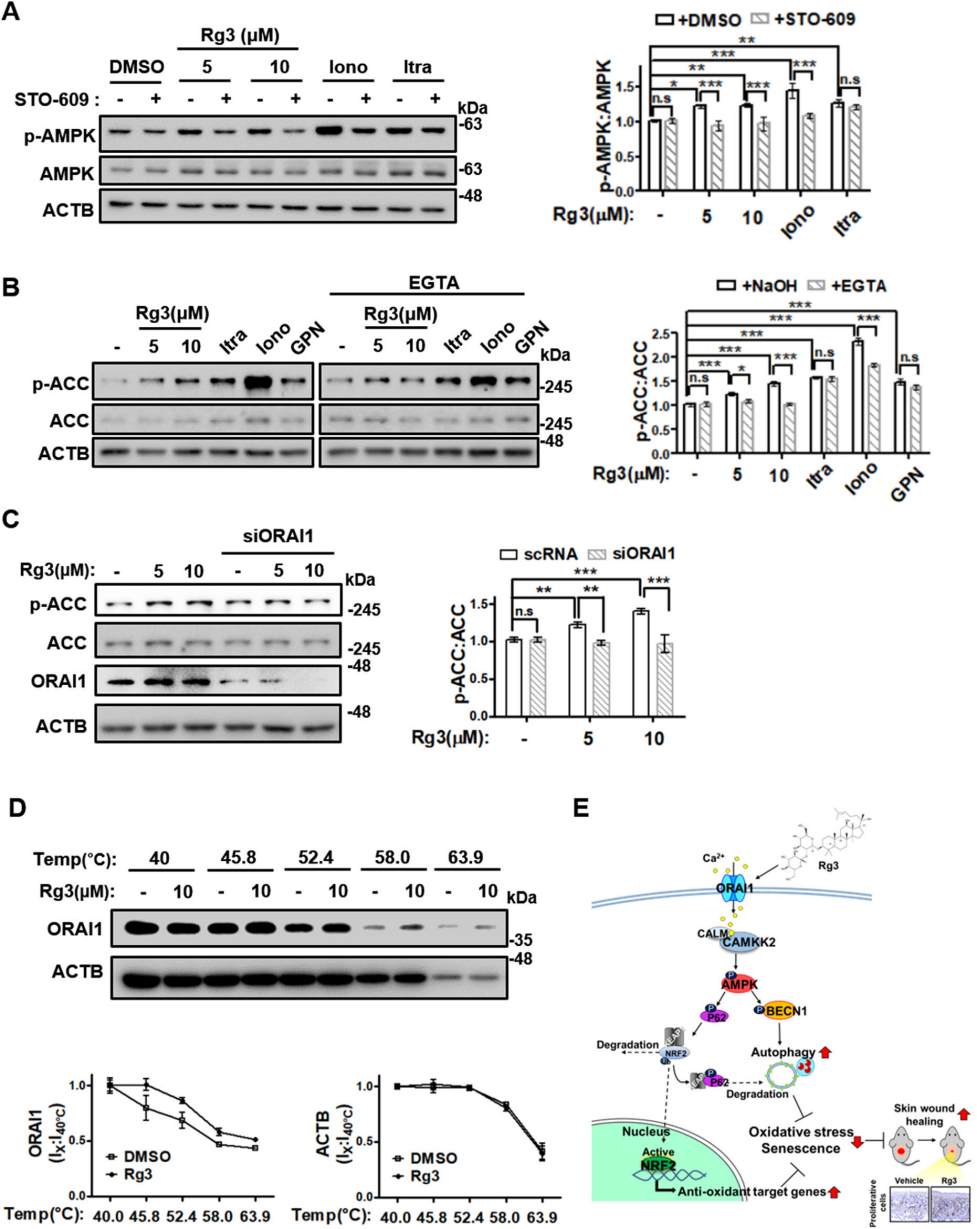
Rg3 promotes extracellular Ca^2+^ ion influx into the cytoplasm by directly perturbing Ca^2+^ ion channel ORAI1 to enhance AMPK signaling. (A) Mid‐old (passage 25) HDFs were treated with Rg3 (5 or 10 μM), 1 μM ionomycin (Iono), or 1 μM itraconazole (Itra) for 1 h with or without pretreatment of 10 μM STO‐609. (B) Mid‐old (passage 24) HDFs were treated with Rg3 (5 or 10 μM), 1 μM Itra, 1 μM Iono, and 50 μM Gly‐Phe‐β‐naphthylamide (GPN) for 1 h with or without pretreatment of 2 mM ethylene glycol tetraacetic acid (EGTA). (C) Old HDFs were transfected with siRNA targeting ORAI1 for 24 h, and then treated with Rg3 (5 or 10 μM) for 1 h. Cell extracts from each experiment (A–C) were subjected to western blotting. Representative images (left) and immunoblot band intensity normalized to ACTB (right). Graphs show the means ± SD (*n*  =  3). (D) Mid‐old (passage 25) HDFs were treated with 10 μM Rg3 for 90 min, and then cellular thermal shift assay analysis was assessed. Representative images (upper) and immunoblot band intensity of each temperature normalized to the band intensity of 40°C. Graphs show the means ± SD (*n*  =  2). Statistical significance was assessed by one‐way ANOVA with Tukey's post‐hoc test. ***p < 0.001; **p < 0.01; *p < 0.05. (E) Schematic illustration summarizing the mechanism by which Rg3 attenuates cellular senescence via inducing AMPK‐mediated autophagy and antioxidant signaling

ORAI1 is a plasma‐membrane‐Ca^2+^ channel, and its physiological role in skin homeostasis has been revealed.^10^ Thus, we hypothesized that ORAI1 can play a role in Rg3‐induced AMPK signaling activation in HDFs. Under the ORAI1 inhibition, using Biochanin A or siORAI1, activation of AMPK signaling by Rg3 was completely abolished (Figures S5B and 4C). To explore the direct binding of Rg3 with ORAI1, the cellular thermal shift assay (CETSA) was applied. Rg3‐treated cells exhibited enhanced thermal stability of ORAI1, but no alterations of ACTB (Figure 4D). These results indicate that Rg3 promotes Ca^2+^ influx into the cells through directly binding and regulating the ORAI1 Ca^2+^ channel in the plasma membrane.

In conclusion, Rg3 was uncovered to have antisenescent activity, and its target protein ORAI1 was identified using CETSA, a label‐free method. Mechanistically, Rg3 promotes AMPK activation by directly regulating ORAI1 to promote Ca^2+^ influx into the cytoplasm, leading to both autophagy and NRF2 activation. Rg3‐induced autophagy and NRF2 antioxidant signaling abolished both replicative senescence and ROS‐induced senescence in HDFs, ultimately promoted rejuvenation in skin aging (Figure 4E). Consequently, this study reports for the first time that the small natural molecule Rg3 can be a useful chemical probe for autophagy‐dependent antisenescence investigation. Furthermore, the coordinative control of stress‐responsive pathways, autophagy and NRF2 signaling, through the direct perturbation of ORAI1 can be a new promising strategy to target and regulate senescence. These findings can help to effectively establish a future approach to treat aging‐related pathologies.

## FUNDING

This work was partly supported by grants from the National Research Foundation of Korea and was funded by the government of the Republic of Korea (MSIP; 2015K1A1A2028365, 2018M3A9C4076477, 2021R1A3B1077371), the Brain Korea 21 Plus Project, and Institute of Convergence Science (ICONS) at Yonsei University. In addition, this study was partly supported by a grant from Korea Basic Science Institute (C180310).

## CONFLICT OF INTEREST

The authors declare no conflict of interest.

## ETHICS APPROVAL AND CONSENT TO PARTICIPATE

All studies involving animals were approved and performed in accordance with the guidelines of the Institutional Animal Care and Use Committee of Korea Basic Science Institute (KBSI‐AEC 1913). These guidelines follow the Guide for Care and Use of Laboratory Animals published by the US National Institutes of Health (The National Academies Press, 8th Edition, 2011).

## AUTHOR CONTRIBUTIONS

D.K., D.W.K., and K‐E.Y. conducted the experiments. D.K., D.W.K., K‐E.Y., H‐Y. H., and J. K. participated in the data analysis. D.K., J‐S.C., and H.J.K. designed the experiments and drafted the manuscript. J‐S.C. and H.J.K. initiated and managed the study. All authors read and approved the final manuscript.

## DATA AVAILABILITY STATEMENT

The data supporting the conclusions of this study are included within the article and its additional file.

## Supporting information

Supporting information
