# Supplementary material for: Activation of Ca2+‐AMPK‐mediated autophagy by ginsenoside Rg3 attenuates cellular senescence in human dermal fibroblasts
Source: Clin Transl Med. 2021 Aug 9;11(8):e521. doi: 10.1002/ctm2.521 (PMC8351519; doi:10.1002/ctm2.521)
Supplement: Supplementary file 1 — Supporting information [file CTM2-11-e521-s001.docx]

SUPPORTING INFORMATION FOR

**Activation of Ca^2+^-AMPK-mediated autophagy by ginsenoside Rg3 attenuates cellular senescence in human dermal fibroblasts**

Dasol Kim^1^, Kyeong Eun Yang^2^, Dong Won Kim^1^, Hui-Yun Hwang^1^, Jinyoung Kim^1^, Jong-Soon Choi^3*^, and Ho Jeong Kwon^1^*

**MATERIALS AND METHODS**

**Cell culture**

Primary HDF cells were cultured in Dulbecco's modified Eagle medium supplemented with 10% bovine serum (v/v) and 1% penicillin/streptomycin (v/v). HDFs were grown to less than 80% confluence in each subculture. To induce replicative senescence, HDFs were subcultured until more than passage number 29. Cell cultures were maintained at pH 7.4 in a humidified incubator at 37°C in 5% (v/v) atmospheric CO_2_. Primary HDF cells were purchased from Coriell Institute for Medical Research (Camden, NJ).

**mRFP-GFP-LC3B plasmid transfection**

Cells were transfected with the mRFP-GFP-LC3B plasmid using lipofectamine LTX transfection reagent (Invitrogen) for 24 h. The cells were treated with drugs for 24 h. Nuclei were stained with 20 mM Hoechst 33342 (Thermo Fisher Scientific) and incubated for 20 min. Next, the cells were fixed with 4% formaldehyde and washed three times with phosphate-buffered saline (PBS). Images were obtained using an LSM880 confocal microscope at 400× magnification, and then, red and green puncta were counted.

**Immunoblotting**

Soluble proteins were harvested from cells using sodium dodecyl sulfate lysis buffer (50 mM Tris-HCl at pH 6.8 containing 10% glycerol, 2% SDS, 10 mM dithiothreitol, and 0.005% bromophenol blue). Equal volumes of proteins were separated using 8% or 12.5% sodium dodecyl sulfate–polyacrylamide gel electrophoresis and transferred to polyvinylidene fluoride membranes (EMD Millipore, Billerica, MA). The blots were then blocked and immunolabeled overnight at 4°C with the following primary antibodies: anti-LC3B, anti-p-ACC, anti-ACC, anti-p-AMPK, anti-AMPK, anti-p-BECN1, anti-BECN1 (Cell Signaling Technology), anti-p53, anti-p21, anti-actin (Abcam), anti–nuclear factor erythroid 2–related factor 2 (NRF2; Santacruz Biotechnology), anti-p62 (BD Biosciences, Franklin Lakes, NJ), anti-p-p62 (MBL), anti–heme oxygenase 1 (HMOX1; Enzo Life Sciences), and anti-protein ORAI1 (Novus). Immunolabeling was visualized using an enhanced chemiluminescence kit (Amersham Life Science, Inc., Amersham, UK) according to the manufacturer’s instructions. Images were quantified using Image Lab (Bio-Rad, Hercules, CA). Actin was used as an internal control. All band intensity was proportional to the amount of target protein on the membrane with the linear range of detection.

**Cellular thermal shift assay**

HDF cells were treated with Rg3, harvested using a scraper, and centrifuged at 300 g for 3 min. The cells were then gently washed with PBS and centrifuged again at 300 g for 3 min. Cells were resuspended in PBS and split into separate pools in polymerase chain reaction tubes (PCR; 8-strip). Cells with each PCR strip were subsequently heat shocked at various temperatures for 4 min, then incubated in 25°C for 3 min. Samples were immediately snap-frozen 4–5 times using liquid nitrogen and a heating block set at 25°C. Each sample was transferred into 1.5 mL tubes, and the cell lysate–containing tubes were centrifuged at 20,000 g for 20 min at 4°C. The soluble supernatant protein fractions were transferred and analyzed by immunoblotting.

**SA-β-gal staining**

Senescence-associated β-galactosidase (SA-β-gal) was analyzed using a SA-β-gal staining kit (Sigma) according to the manufacturer’s instructions. Briefly, cells were seeded at a density of 2×10^4^ cells/well in 12-well plates. Rg3 (S)-treated senescent HDFs were first fixed for 10 min at room temperature in fixation buffer. Cells were then washed with PBS and stained with β-gal staining solution for 16 h at 37°C without CO_2_. Stained cells were detected under a microscope at ×100 magnification. The degree of SA-β-gal positive cells was counted from five randomly chosen fields.

**ROS measurement**

Intracellular ROS were detected using the fluorescent probe 5-(and 6)-carboxy-2′,7′-dichlorodihydrofluorescein diacetate (DCF-DA) (Sigma). HDFs were seeded in 96-well plates, and senescent HDFs were treated with Rg3 for 48 h. Subsequently, cells were incubated with 10 μM DCF-DA in darkness for 30 min at 37°C. After cells were washed with PBS, green fluorescence of DCF-DA was measured at excitation wavelength 485 nm and at emission wavelength 535 nm using a TriStar^2^ LB 942 multimode microplate reader (Berthold Technologies).

**Immunohistochemistry in an aging mouse model**

In C57BL/6 mice aged 4 or 18 months, dorsal hairs were removed. Wounds were created on the backs of mice (n = 6 in each group) the day after hair removal. Rg3 (100 μM) or the vehicle control dissolved in saline was applied to the wounding site, and the wound was directly covered with dressing. This application was repeated every 2 days for a week. Skin tissues were harvested and embedded in paraffin. Paraffin blocks were cut into 4-µm thick sections. After deparaffinization and rehydration, endogenous peroxidase activity was blocked by incubation with 3% hydrogen peroxide (H_2_O_2_) for 15 min. Antigen was retrieved by microwave pressure cooking in citrate buffer (10 mM citric acid; pH, 6.8) for 9 min. The sections were blocked for 1 h at room temperature with 3% bovine serum albumin and incubated with primary anti–proliferating cell nuclear antigen (Cell Signaling Technology) and anti-Ki-67 (Cell Signaling Technology). The antigen-antibody complex was detected using the Dako REAL™ Envision™ detection system, peroxidase/DAB+, and rabbit/mouse (Dako). Immunohistochemically stained skin sections were counterstained with hematoxylin (Merck). The stained slides were scanned using Imager Z2 (Carl Zeiss) with TissueFaxs version 4.2 software (TissueGnostics), and quantitative image data analysis was performed using HistoQuest software (TissueGnostics).

**Statistical analysis**

All data are expressed as the means ± standard deviation (SD) or the means ± standard error of the mean (SEM), as determined using GraphPad Prism (version 5.00 for Windows; GraphPad Software, Inc., San Diego, CA). Quantitative data were obtained from at least three independent experiments unless otherwise noted. Statistical analyses were performed using unpaired two-tailed Student’s *t*-test or one-way ANOVA with Tukey’s post-hoc test and a *P*-value of less than 0.05 was considered statistically significant (*indicates *P*< 0.05; **indicates *P*< 0.01; ***indicates *P*< 0.001).

**DISCUSSION**

This study showed that the natural compound Rg3 attenuates cellular senescence and improves aging decline in skin tissue without cytotoxicity (**Figure S6**). The inflammatory response of Rg3 treatment in skin tissue was not determined in this study since there was a report that Rg3 demonstrated the anti-inflammatory effect via inhibition of NFκB pathway^1^. Rg3 decreased the expression of the aging marker TP53 and CDKN1A as well as the SA-β-gal staining in HDF cells. In addition, Rg3 increased the expression levels of PCNA and Ki-67 in old skin tissue by IHC staining. The expression of p53 and p21 in aged mouse skin was up-regulated as reported previously^2^. The senescent cells% of aged mice tissue are known to be larger than non-senescent cells^3^. Wound healing is an intricately intertwined *in vivo* process with numerous cellular proliferation regulators^4^. Ki-67 and proliferating cell nuclear antigen (PCNA) are known to be cellular markers for cell proliferation. Ki-67 is expressed in the nucleus of proliferating cells not only in S phase, but also in G1, G2 and M phases of the cell cycle^5^, while its expression keeps constant in the resting phase. Expression of PCNA increases in the late G1 phase. After peaking in mid-S phase, it returns to initial level in late S phase^6^.

Senescence-associated secretory phenotype (SASP) is another key phenotype of senescent cells, which involves production and secretion of cytokines that trigger environmental senescence and inflammation. NFκB pathway has been reported to control SASP thereby enhancing cellular senescence and chemosensitivity^7^. In addition, Hou *et al*. previously reported that Rg3 attenuated ROS-induced NFκB activation and SASP in astrocytes^8^, and Gum *et al.* has reported that Rg3 enhances NRF2 activation^9^, as in line with this study, which recently revealed as controversial regulatory factor of cellular inflammation and senescence^10-12^. Although this study did not fully address those issues, regulatory role of NRF2 signaling by Rg3 in ROS-induced inflammation and SASP should be identified in the following study.

Rg3 regulates senescence through autophagy induction, especially in an AMPK-dependent manner (**Figure 3C-G and S7**). These results are in line with a report showing that AMPK activation protected cells from oxidative stress–induced senescence via enhancing autophagy flux^13^. These data are also consistent with a report that pharmacological induction of LKB1-AMPK signaling inhibited senescence through autophagy activation in vascular smooth muscle cells^14^. Proper regulation of AMPK is pivotal in cellular life because AMPK coordinates large signaling networks such as energy metabolism, autophagic degradation, and stress resistance in cells^15^. With aging, the sensitivity of AMPK activation declines, leading to autophagy attenuation and stress induction in aged cells. Therefore, the pharmacological activation of AMPK in aged cells can be a key strategy to slow their decline in function due to aging.

Our data also demonstrated that Rg3-induced AMPK signaling activation is mediated by Ca^2+^ regulation, indicating the activation of CAMKK2-AMPK signaling rather than LKB1-AMPK signaling. A previous report found that regulation of the Ca^2+^ ion is critical to control maintenance of skin function^16^. Belkacemi A. *et al.* revealed that enhanced Ca^2+^ concentration in the cytoplasm by Cavβ deficiency promotes collagen secretion and fibroblast migration, leading to acceleration of the wound healing process^17^. However, senescent fibroblasts exhibit reduced collagen secretion, extracellular matrix production^18^, and cell migration^19^. Therefore, CAMKK2-AMPK activation by intracellular Ca^2+^ upregulation can be a strategy to prevent cellular senescence in fibroblasts.

This study showed that the Ca^2+^ channel ORAI1 directly binds to Rg3 through applying CETSA approach. CETSA, a label free method, has been developed in advantage of ligand-induced thermal stabilization of target proteins, meaning that melting temperature of proteins will change upon ligand interaction. Hence, by heating vehicle treated or small molecule treated samples (derived from intact cells or protein lysates) to different temperatures, and quantifying proteins in the ‘soluble (non-melting)’ fraction, it is possible to detect altered protein stability upon small molecule interaction. This approach has been applied to investigate specific target candidates of bioactive small molecule with a high efficiency^20,21^.

ORAI1 is required for Rg3-induced AMPK activation. Thus, ORAI1 may be a relevant target protein of Rg3 for antisenescence activity. Previous studies have reported the positive role of ORAI1 in skin homeostasis such as keratinocyte differentiation, immune response, atopic dermatitis prevention, and hair loss prevention^22-24^. However, regarding senescence, more exploration is required to define the target relevancy of ORAI1 to Rg3-induced biological activity in fibroblasts. For example, contrary to our study, Xu Y. *et al.* revealed that the overexpression of ORAI1 promotes cellular senescence in prostate cancer. Although our study demonstrated that Rg3 directly bound to ORAI1, and promoted Ca^2+^ uptake through the ion channel, target relevancy should be investigated in future studies, not only for ORAI1 but also for other Ca^2+^ channels such as transient receptor potential channels and voltage-gated Ca^2+^ channels located in the plasma membrane.

**SUPPLEMENTAL FIGURES**


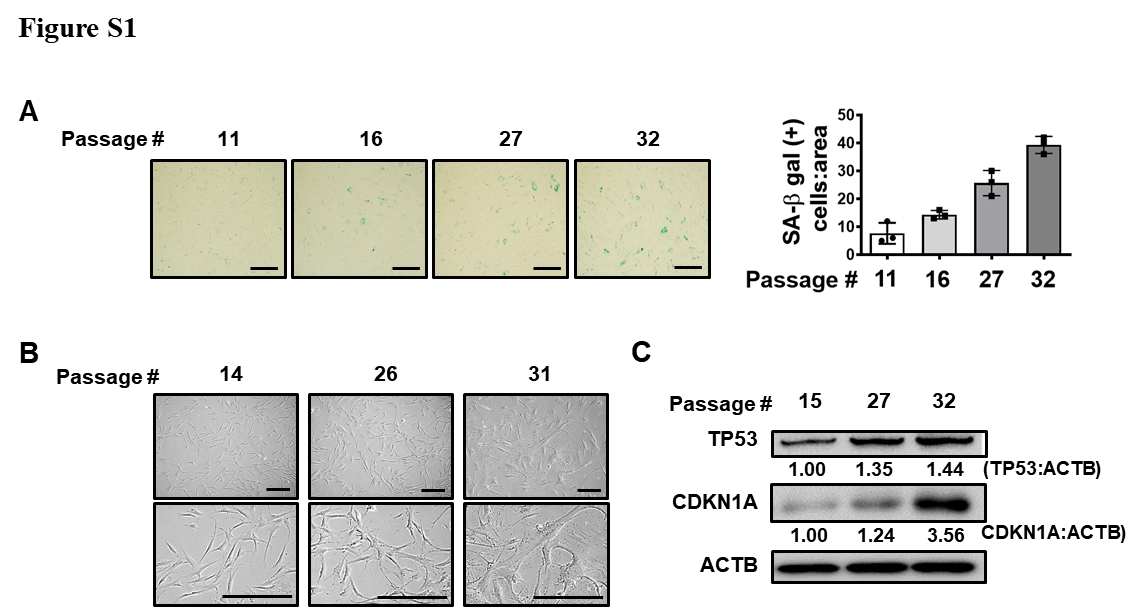


**FIGURE S1. Senescent characteristics in HDFs.** (A) HDF cells in different passage numbers were processed for SA-β-gal assay. Representative images (left) and cell counting per fields (right). Graph shows mean ± SD (n=3). Scale bar, 200 μm. (B) Morphology analysis of HDF cells in different passage numbers by microscopy. Scale bar, 200 μm. (C) HDF cell extracts from different passage numbers were subjected to western blotting. Numbers indicates immunoblot band intensity normalized to ATCB.


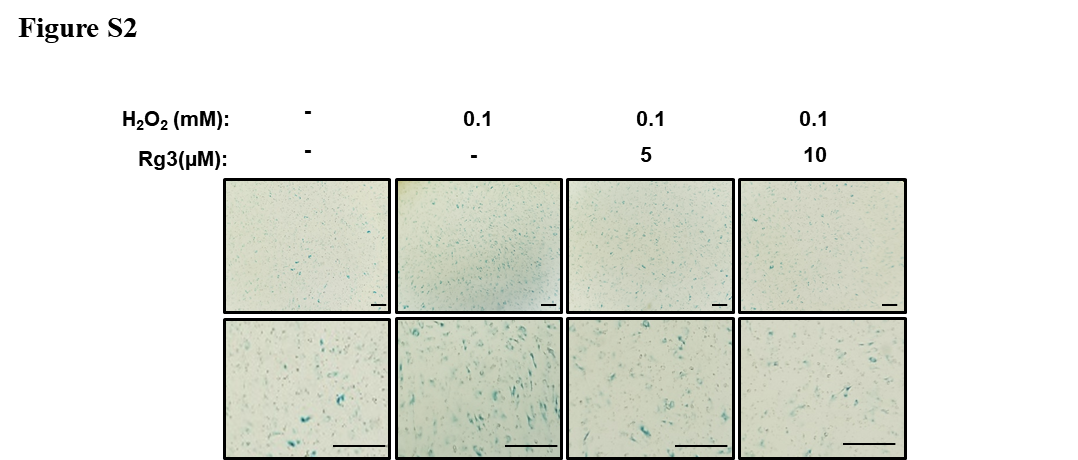


**FIGURE S2. Rg3 reduces oxidative stress-induced senescence in HDFs.** Young HDF cells were treated with 0.1 mM H_2_O_2_ with or without Rg3 as indicated concentration for 48 h. Cells were processed for SA-β-gal assay. Representative images are shown. Scale bar, 500 μm. Graph showing the number of cell counts per field with means ± SD (n = 4) is provided in main Fig 1F.

**
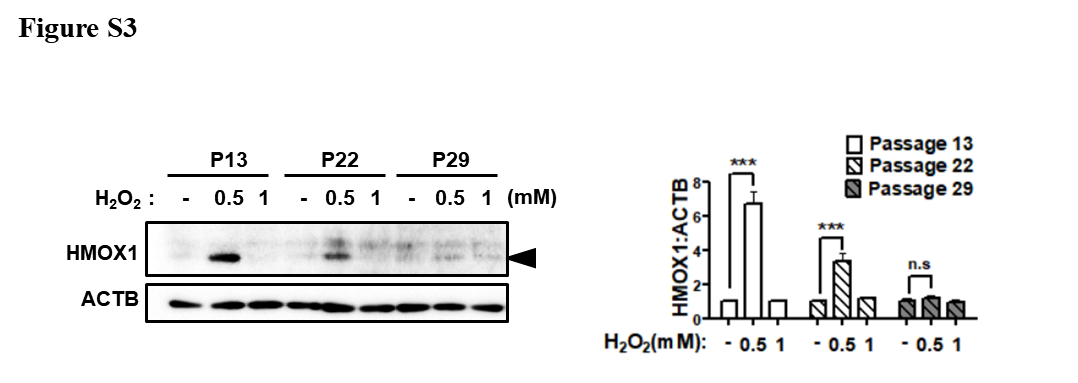
**

**FIGURE S3. Oxidative stress responsive capability in HDFs.** HDF cells in different passage numbers were treated with H_2_O_2_ for 4h, then subjected to western blot analysis. Representative images (left) and immunoblot band intensity normalized to ACTB (right). Graph shows mean ± SD (n=2). Statistical significance was assessed by one-way ANOVA with Tukey’s post-hoc test. ****P*<0.001; ***P*<0.01; **P*<0.05.


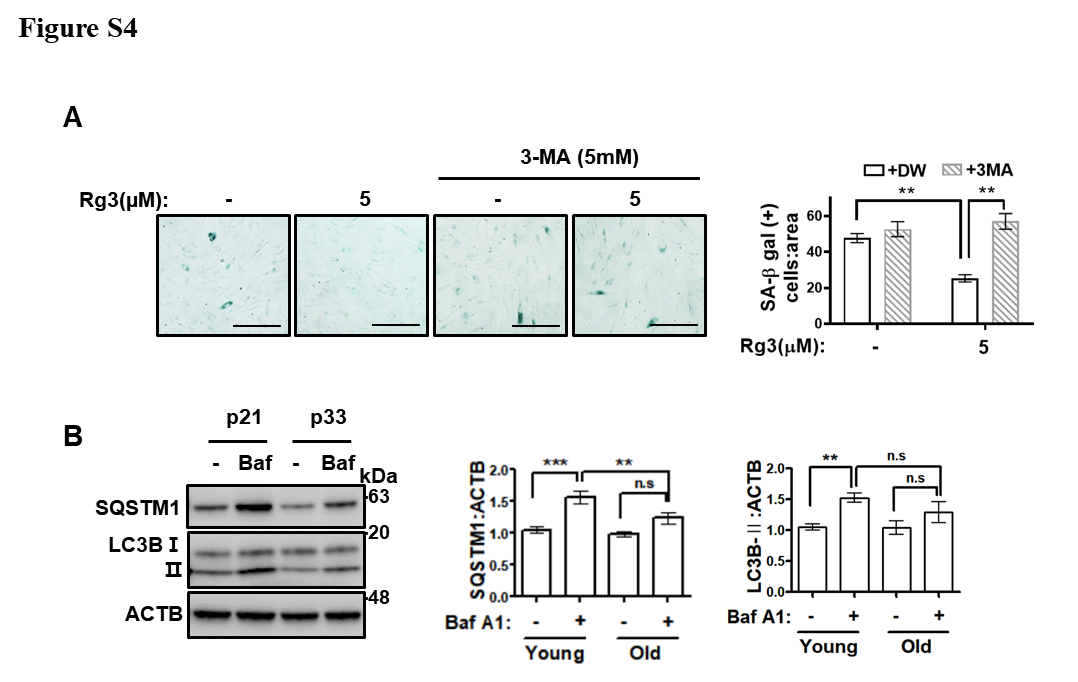


**FIGURE S4. Rg3 requires autophagy for anti-senescence activity.** (A) Old (passage 30) HDF cells were treated with 5 μM Rg3 with or without 5 mM 3-MA for 48h, then processed SA-β-gal assay. Representative images (left) and cell counting per fields (right). Graph shows mean ± SD (n=3). Scale bar, 200 μm. (B) Young (passage 21) and old (passage 33) HDFs were treated with 50 nM bafilomycin A1 for 4 h. Cell extracts were subjected to immunoblotting. Representative images (left) and immunoblot band intensity normalized to ACTB (right). The graphs show the means ± SD (n = 3). Statistical significance was assessed by one-way ANOVA with Tukey’s post-hoc test. ****P*< 0.001; ***P*< 0.01; **P*< 0.05.


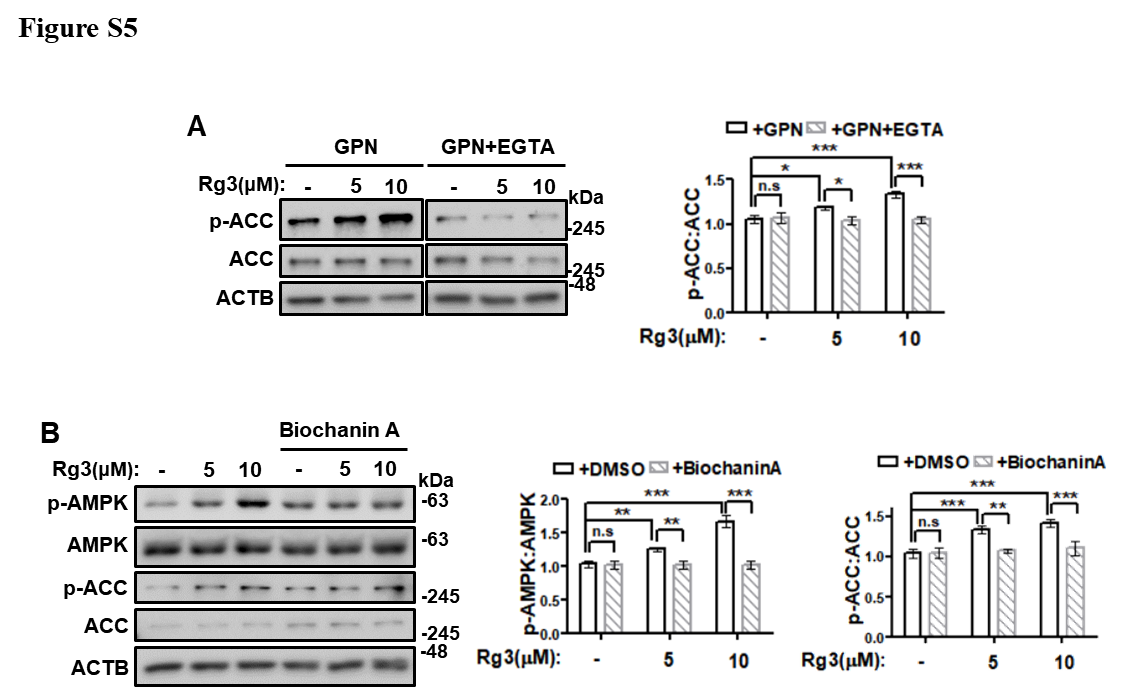


**FIGURE S5. Rg3 requires plasma membrane-located ORAI1 to enhance AMPK activation.** (A) Young (passage 18) HDFs were treated with Rg3 (5 or 10 μM) for 1 h with or without pretreatment of 50 μM GPN or 50 μM GPN + 2 mM EGTA. (B) Mid-old (passage 26) HDFs were treated with Rg3 (5 or 10 μM) for 1 h with or without pretreatment of 20 μM biochanin A. Cell extracts from each experiment (A-B) were subjected to immunoblotting. Representative images (left) and immunoblot band intensity normalized to ACTB (right). Graphs show the means ± SD (n = 3). Statistical significance was assessed by one-way ANOVA with Tukey’s post-hoc test. ****P*< 0.001; ***P*< 0.01; **P*< 0.05.


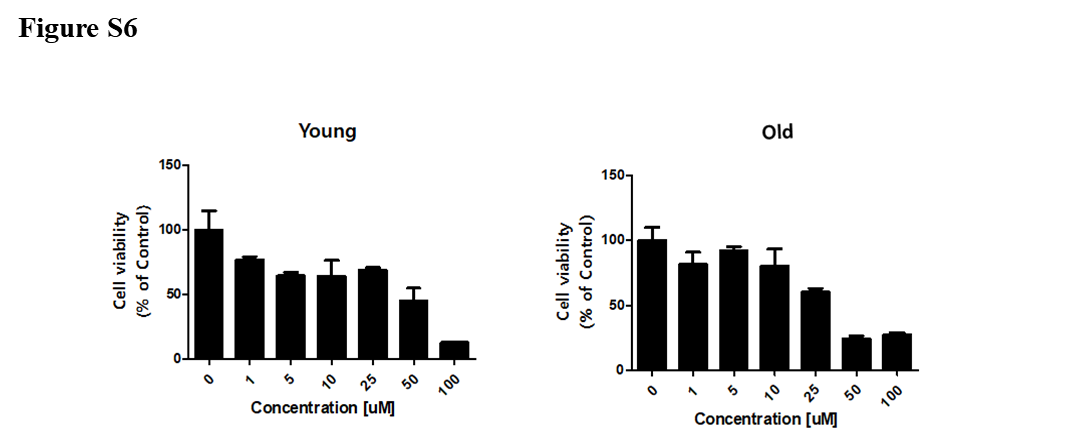


**FIGURE S6. No significant cytotoxicity is observed up to 10 uM in young and old HDFs pretreated with Rg3.** Young and old HDFs were treated with Rg3 as indicated concentration for 48 h. Cell cytotoxicity was measured via trypan blue assay.


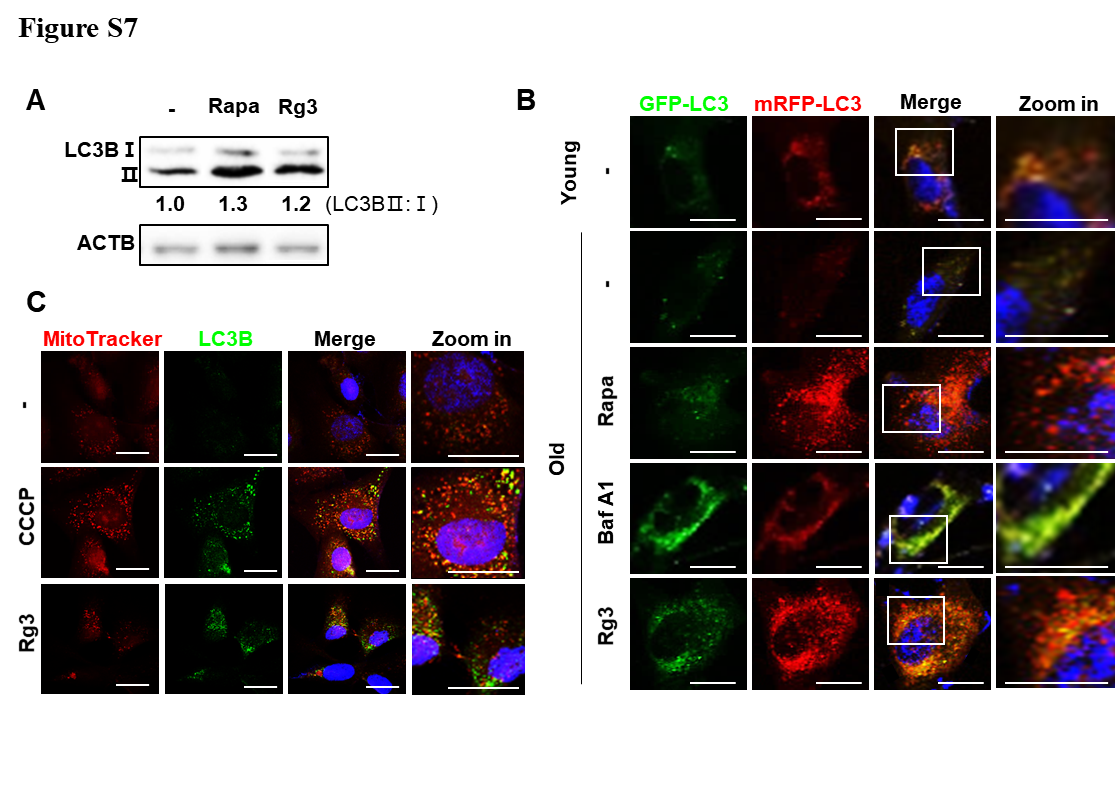


**FIGURE S7. Rg3 enhances conventional macro autophagy in HDFs.** (A) Old HDFs were treated with DMSO vehicle, Rapa (5 μM), or Rg3 (5 μM) for 24 h. Cell lysates were subjected to immunoblotting. The number below the blot indicates LC3B-Ⅱ band intensity normalized to LC3B-Ⅰ. (B) Young and old HDFs were transfected with double-tagged GFP-mRFP-LC3 for 24 h, then treated with DMSO vehicle, Rapa (5 μM), Baf A1 (10 nM), or Rg3 (5μM) for 24 h. Cells were fixed, stained with Hoechst (blue). Representative images of cells under confocal microscopy (upper) showing yellow (autophagosome) and red (autolysosome) puncta in the cells. Scale bar, 20 μm. (C) Mid-old (passage 27) HDFs were treated with DMSO vehicle, CCCP (20 μM), or Rg3 (5 μM) for 24 h. Cells were stained with mitotracker (mitochondria, red), and immune-stained with LC3B antibody (autophagosome, green). Confocal analysis was assessed. Representative images are shown. Scale bar, 20 μm.


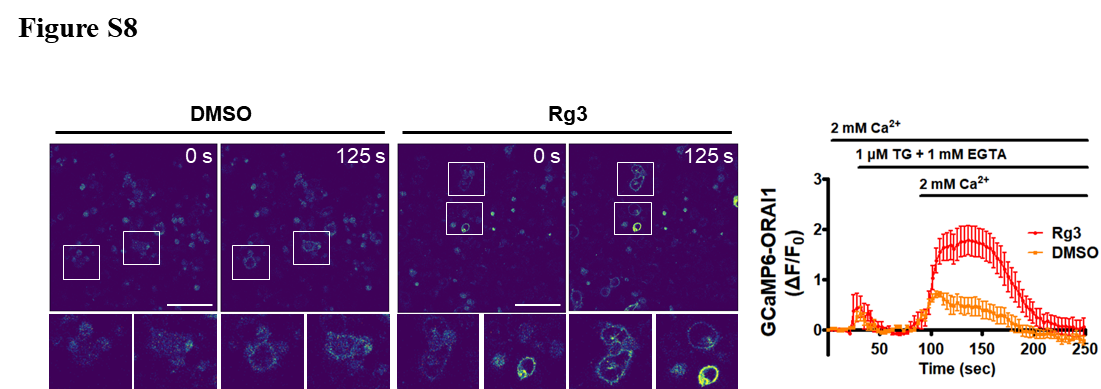


**FIGURE S8. Rg3 enhances conventional macro autophagy in HDFs.** GCaMP6-ORAI1 transfected HeLa cells were pre-treated with Rg3 (10 μM) for 30 min, then subjected to GCaMP imaging analysis using confocal microscopy. Representative images (left) and GCaMP6 fluorescence quantification graph (right) are shown. Scale bar, 100 μm.


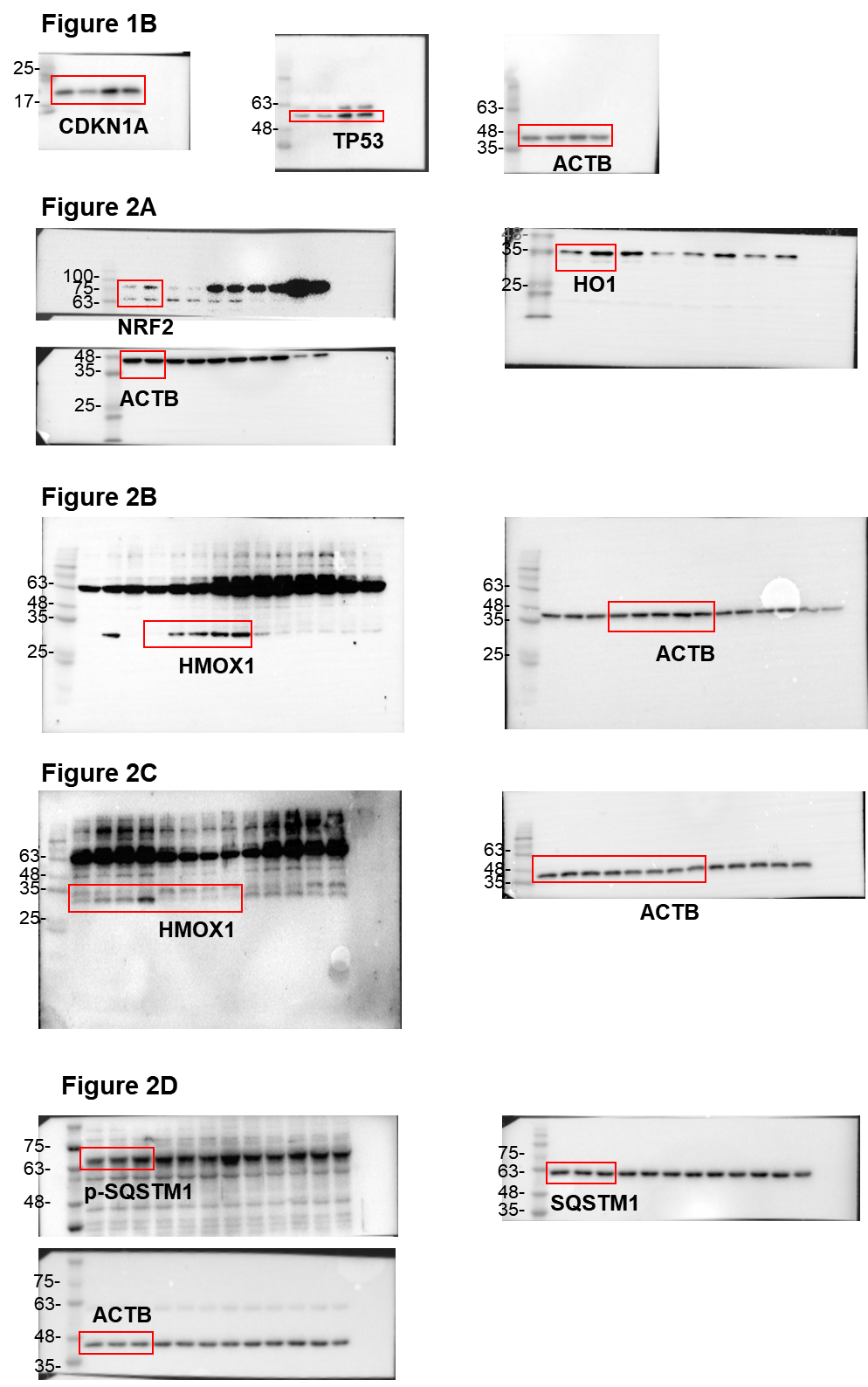


**FIGURE S6. Original western blots shown in Figure 1 and 2. Each figures corresponds to the western blots in the indicated Figure number.**


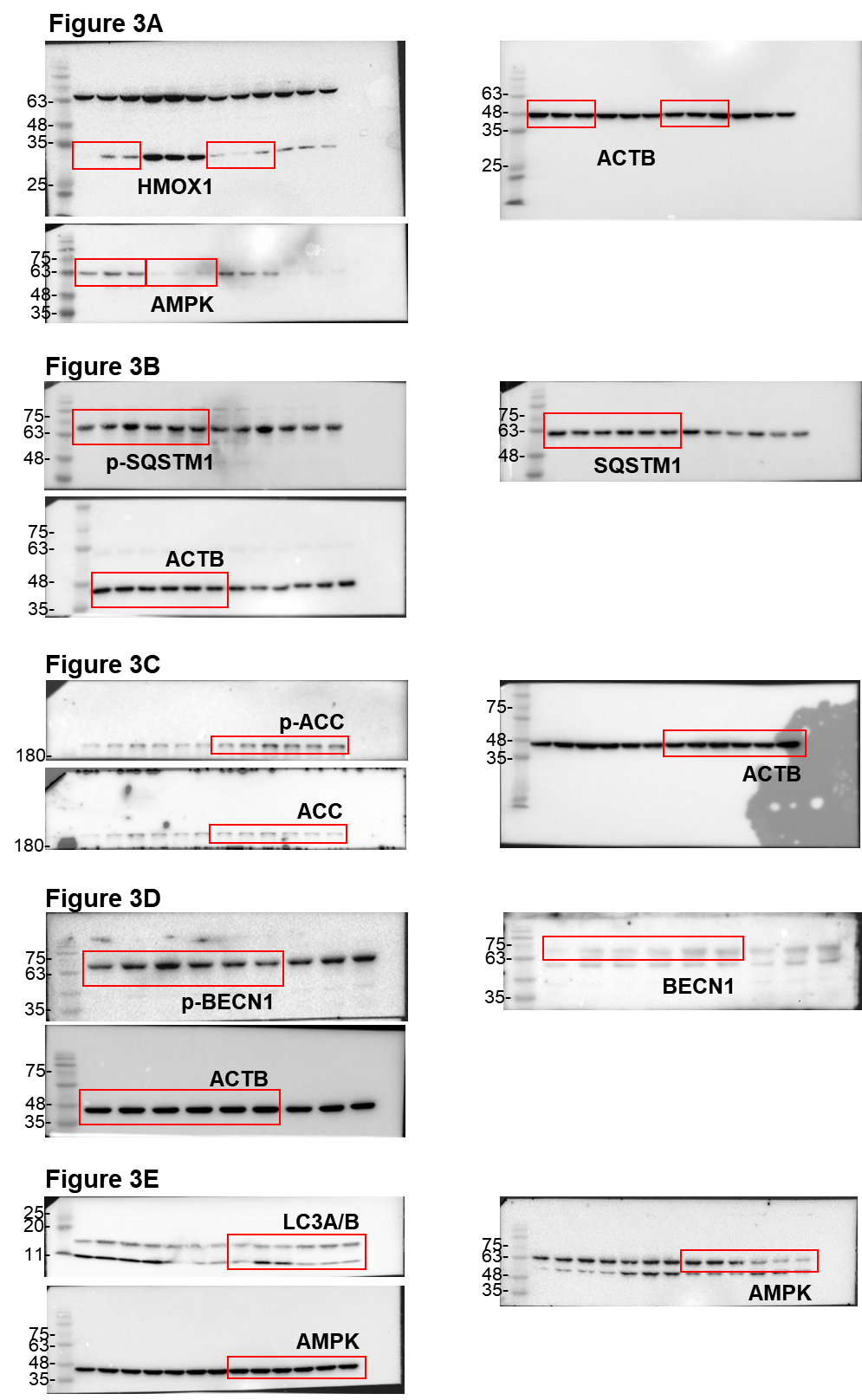


**FIGURE S7. Original western blots shown in Figure 3. Each figures corresponds to the western blots in the indicated Figure number.**


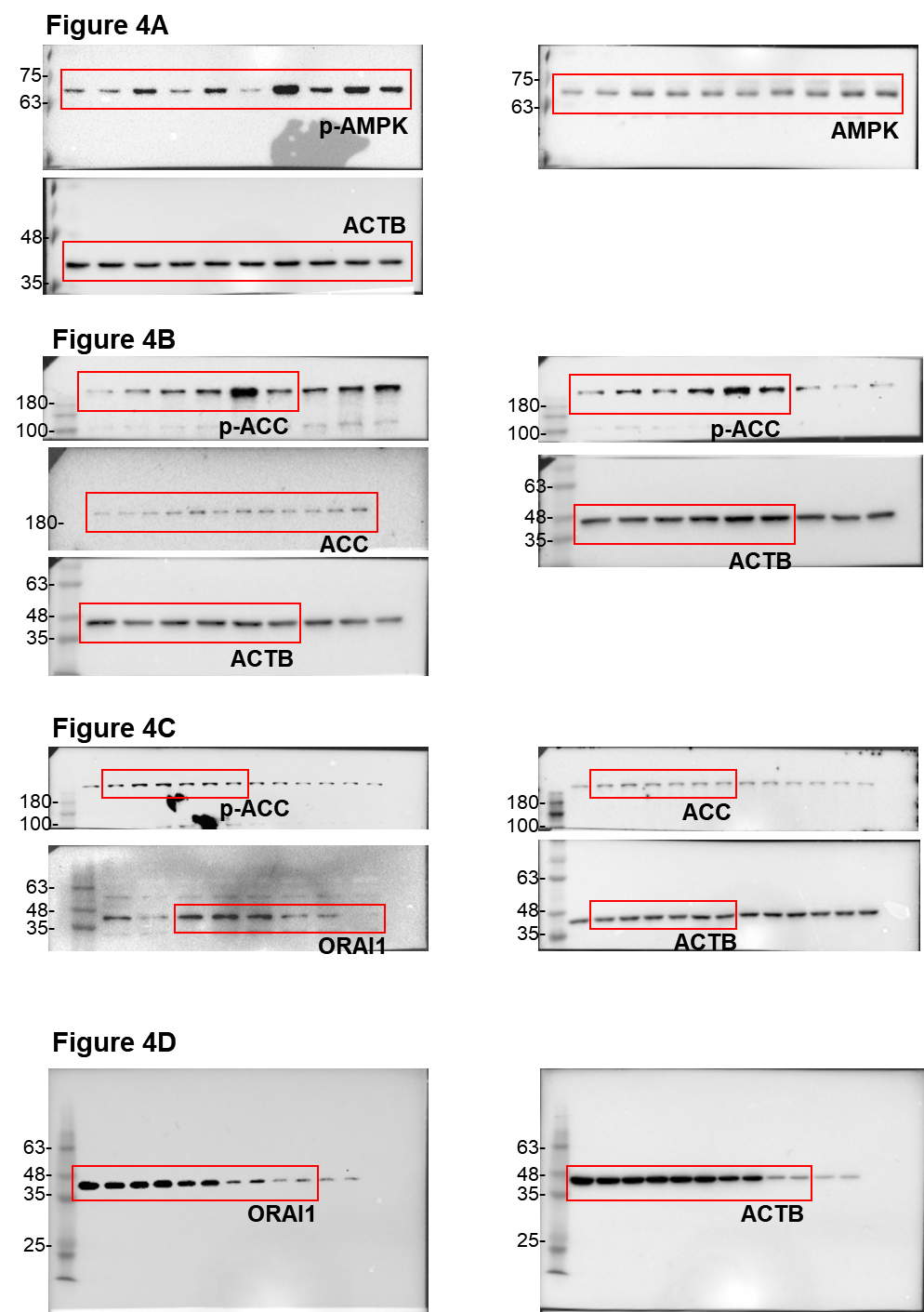


**FIGURE S8. Original western blots shown in Figure 4. Each figures corresponds to the western blots in the indicated Figure number.**

**
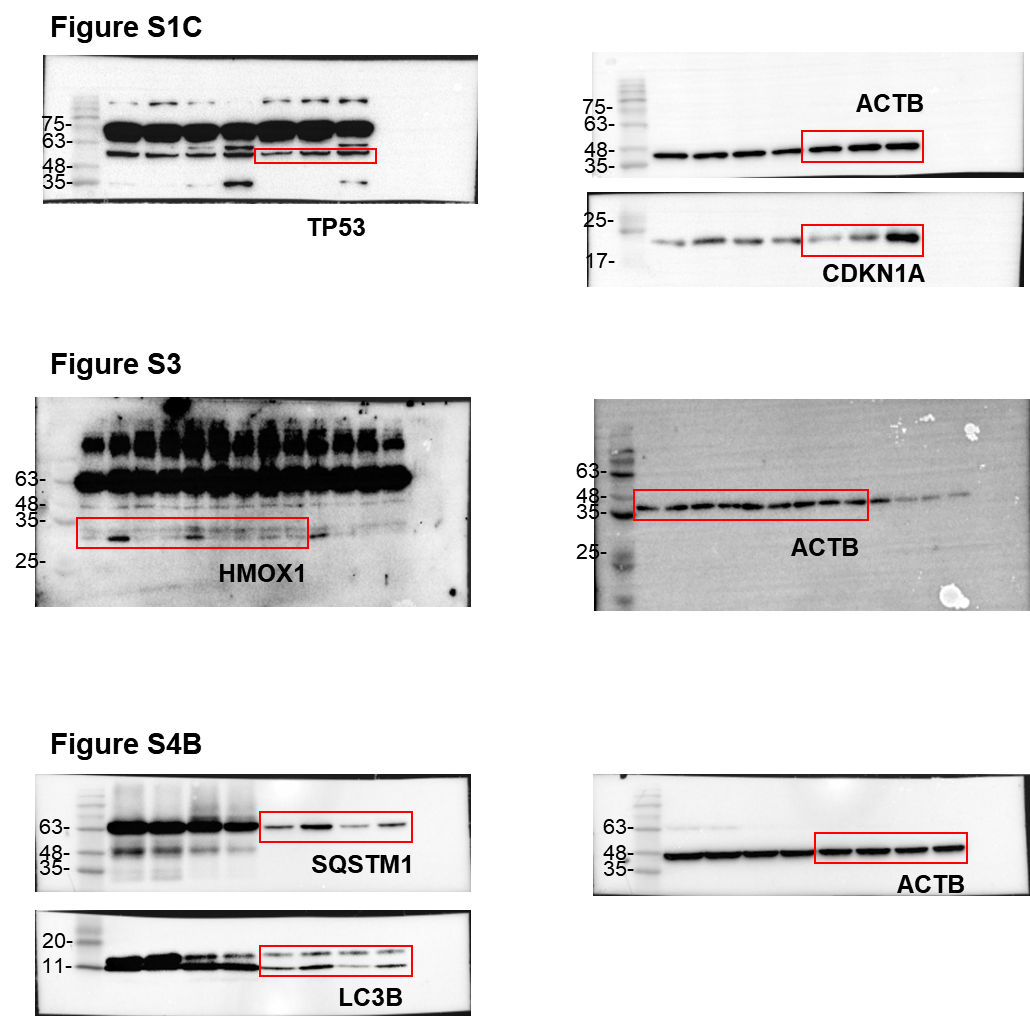
**

**FIGURE S9. Original western blots shown in Figure S1, S3 and S4. Each figures corresponds to the western blots in the indicated Figure number.**

**
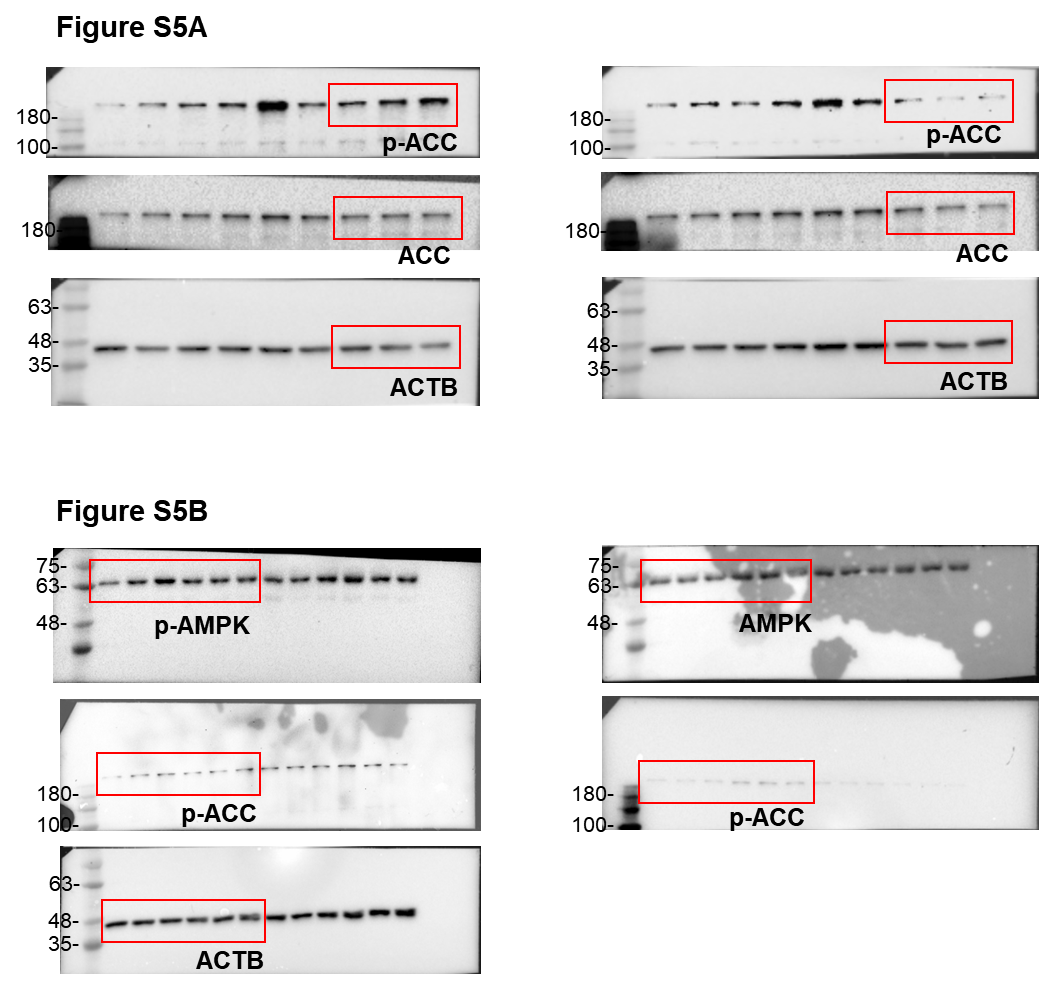
**

**FIGURE S10. Original western blots shown in Figure S5. Each figures corresponds to the western blots in the indicated Figure number.**

**REFERENCE**

1. Lee, I.S., *et al.* Anti-inflammatory effects of ginsenoside Rg3 via NF-κB pathway in A549 cells and human asthmatic lung tissue. *J Immunol Res.* **2016**, 7521601 (2016).

2. Baker, D.J., Weaver, R.L. & van Deursen, J.M. p21 both attenuated and drives senescence and aging in BubR1 progeroid mice. *Cell Rep.* **3**, 1164-1174 (2013).

3. Biran, A. et al. Quantitative identification of senescent cells in aging and disease. *Aging Cell.* **16**, 661-671 (2017).

4. Onuma, S., Mastui, C. & Morohashi, M. Quantitative analysis of the proliferation of epidermal cells using a human skin organ culture system and the effect of DbcAMP using markers of proliferation (BrdU, Ki-67, PCNA). *Arch Dermatol Res.* **293**, 133–138 (2001).

5. Gerdes, J., *et al.* Growth fractions in breast cancers determined in situ with monoclonal antibody Ki-67*. J Clin Pathol.* **39**, 977–980 (1986).

6. Celis, J.E. & Celis, A. Cell cycle-dependent variations in the distribution of the nuclear protein cyclin proliferating cell nuclear antigen in cultured cells: Subdivision of S phase. *Proc Natl Acad Sci U S A.* **82**, 3262-3266 (1985).

7. Chien, Y.*, et al*. Control of the senescence-associated secretory phenotype by NF-kappaB promotes senescence and enhances chemosensitivity. *Genes Dev*. **25**, 2125-2136 (2011).

8. Hou, J., Kim, S., Sung, C. & Choi, C. Ginsenoside Rg3 Prevents Oxidative Stress-Induced Astrocytic Senescence and Ameliorates Senescence Paracrine Effects on Glioblastoma. *Molecules*. **22**, (2017).

9. Gum, S.I. & Cho, M.K. The amelioration of N-acetyl-p-benzoquinone imine toxicity by ginsenoside Rg3: the role of Nrf2-mediated detoxification and Mrp1/Mrp3 transports. *Oxid Med Cell Longev*. **2013**, 957947 (2013).

10. Hiebert, P.*, et al*. Nrf2-Mediated Fibroblast Reprogramming Drives Cellular Senescence by Targeting the Matrisome. *Dev Cell*. **46**, 145-161 e110 (2018).

11. Ahmed, S.M., Luo, L., Namani, A., Wang, X.J. & Tang, X. Nrf2 signaling pathway: Pivotal roles in inflammation. *Biochim Biophys Acta Mol Basis Dis*. **1863**, 585-597 (2017).

12. Yuan, H., Xu, Y., Luo, Y., Wang, N.X. & Xiao, J.H. Role of Nrf2 in cell senescence regulation. *Mol Cell Biochem*. **476**, 247-259 (2021).

13. Han, X.*, et al*. AMPK activation protects cells from oxidative stress-induced senescence via autophagic flux restoration and intracellular NAD(+) elevation. *Aging Cell*. **15**, 416-427 (2016).

14. Lee, K.Y., Kim, J.R. & Choi, H.C. Genistein-induced LKB1-AMPK activation inhibits senescence of VSMC through autophagy induction. *Vascul Pharmacol*. **81**, 75-82 (2016).

15. Salminen, A. & Kaarniranta, K. AMP-activated protein kinase (AMPK) controls the aging process via an integrated signaling network. *Ageing Res Rev*. **11**, 230-241 (2012).

16. Lee, S.E. & Lee, S.H. Skin Barrier and Calcium. *Ann Dermatol*. **30**, 265-275 (2018).

17. Belkacemi, A.*, et al*. IP3 Receptor-Dependent Cytoplasmic Ca(2+) Signals Are Tightly Controlled by Cavbeta3. *Cell Rep*. **22**, 1339-1349 (2018).

18. Mellone, M.*, et al*. Induction of fibroblast senescence generates a non-fibrogenic myofibroblast phenotype that differentially impacts on cancer prognosis. *Aging (Albany NY)*. **9**, 114-132 (2016).

19. Bian, X.*, et al*. Regenerative and protective effects of dMSC-sEVs on high-glucose-induced senescent fibroblasts by suppressing RAGE pathway and activating Smad pathway. *Stem Cell Res Ther*. **11**, 166 (2020).

20. Martinez Molina, D.*, et al*. Monitoring drug target engagement in cells and tissues using the cellular thermal shift assay. *Science*. **341**, 84-87 (2013).

21. Jafari, R.*, et al*. The cellular thermal shift assay for evaluating drug target interactions in cells. *Nat Protoc*. **9**, 2100-2122 (2014).

22. Numaga-Tomita, T. & Putney, J.W. Role of STIM1- and Orai1-mediated Ca2+ entry in Ca2+-induced epidermal keratinocyte differentiation. *J Cell Sci*. **126**, 605-612 (2013).

23. Gwack, Y.*, et al*. Hair loss and defective T- and B-cell function in mice lacking ORAI1. *Mol Cell Biol*. **28**, 5209-5222 (2008).

24. Chang, W.C.*, et al*. ORAI1 genetic polymorphisms associated with the susceptibility of atopic dermatitis in Japanese and Taiwanese populations. *PLoS One*. **7**, e29387 (2012).
